# Oscar Oldberg

**Published:** 1913-04

**Authors:** 


Oscar Oldberg, Emeritus Dean of the Northwestern University School of Pharmacy, died February 27, in Pasadena. He was born in Sweden January 22, 1846. Having come to America as Vice-Consul of Sweden in Memphis, he was appointed Chief Clerk and Medical Purveyor of the U. S. Marine Hospital (now Public Health) Service, in 1874. As Dean of the National College of Pharmacy in Washington, he introduced the metric system. For three decades he was an active member of the Committee of Revision of the U. S. Pharmacopoeia. Few men have done more to perfect the Materia Medica, and this country owes much to his sagacity and industry.

